# Human anterior chamber angle development without cell death or macrophage involvement

**Published:** 2008-12-26

**Authors:** Beeran Meghpara, Xin Li, Hiroshi Nakamura, Ahsan Khan, Bassem A. Bejjani, Shan Lin, Deepak P. Edward

**Affiliations:** 1 College of Medicine, University of Illinois at Chicago, Chicago, IL; 2 Department of Pathology, University of Michigan, Ann Arbor, MI; 3 Department of Ophthalmology, Summa Health System, Akron, OH; 4 Department of Ophthalmology, Northeastern Ohio Universities College of Medicine and Pharmacy, Akron, OH; 5 Kaiser Permanente Health Systems, Los Angeles, CA; 6 Washington State University Spokane, Spokane, WA; 7 University of California, San Francisco, CA

## Abstract

**Purpose:**

The iridocorneal angle in the mammalian eye including the trabecular meshwork (TM) develops from undifferentiated mesenchyme/neural crest between the iris root and cornea. The precise mechanisms underlying anterior angle development are unclear, and the contribution of cell death and phagocytic resorption by macrophages in angle development is controversial. In this study, we examined the human anterior chamber angle during various stages of development for evidence of cell death and phagocytic resorption.

**Methods:**

Eyes from the human fetus (F) of 7, 8, 9, 10, 11, 13, 15, 18, 19, 21, 22, 23, and 27 weeks as well as eyes from 5- and 11-month-old children and donors 24, 48, and 67 years of age were obtained. Formalin-fixed and paraffin-embedded sections were examined by hematoxylin and eosin (H&E) staining. Immunohistochemistry was performed using polyclonal antibodies against CD68. Terminal deoxynucleotidyl transferase dUTP nick end labeling (TUNEL) labeling was also performed to evaluate cell death.

**Results:**

By light microscopy, the development of human angle structures appeared to progress as previously described. Histological evidence of cellular death or resorption by macrophages was not observed. Furthermore, the chamber angle tissues did not stain with CD68 at any stage of development. Few CD68 positive cells were observed in the iris stroma and the anterior ciliary body between fetal weeks 10 and 18 (F10w and F18w). TUNEL labeled nuclei were not detected in the anterior chamber angle in any fetal or infant eyes. By contrast, TUNEL positive nuclei in TM cells were observed in the examined adult donor specimens.

**Conclusions:**

The results suggest that at the time points examined, neither cell death nor phagocytic resorption with macrophages appear to play a role in the development of the human anterior chamber angle.

## Introduction

The iridocorneal angle in the mammalian eye forms between the root of the iris and cornea. The trabecular meshwork is a prominent component of the anterior chamber angle. It consists of beams of extracellular matrix with channels interspersed between them, which lead to Schlemm’s canal for drainage of the aqueous humor from the eye [1]. Abnormal development of the anterior chamber angle is associated with elevated intraocular pressure in the spectrum of developmental glaucoma [2].

The anterior chamber angle is initially packed with a dense collection of mesenchymal/neural crest cells. As development proceeds, channels form between trabecular beams, which provide a conduit for aqueous humor outflow [3]. Several studies have attempted to explain the mechanism by which the anterior chamber angle forms [4-7]. However, the precise mechanism of anterior chamber angle development remains controversial. One theory suggests that cellular differentiation and reorganization of the original mass of mesenchymal/neural crest cells result in the formation of mature meshwork of beams and spaces [6]. In contrast, others have reported that cell death and/or resorption were observed during intertrabecular space formation [8,9]. The majority of these studies were conducted on various animal species using light and electron microscopy. One recent report used immunolabeling to detect cell death in the developing anterior angle of mice. However, cell death was not observed [10]. In the human eye studies, cell death and/or resorption were evaluated only by their histological appearance and not by molecular markers.

In this study, we examined human eye specimens to determine if cellular death or phagocytic resorption by macrophages was involved in the development of the anterior chamber angle using conventional light microscopy, immunohistochemistry, and Terminal deoxynucleotidyl transferase dUTP nick end labeling (TUNEL) assay.

## Methods

Eyes from human fetuses of 7, 8, 9, 10, 11, 13, 15, 18, 19, 21, 22, 23, and 27 weeks post-conception (w) as well as eyes from 5- and 11-month-old children and donors 24, 48, and 67 years of age were obtained from the University of California at San Francisco (San Francisco, CA), University of Seattle tissue bank (Seattle, WA), University of Illinois at Chicago (Chicago, IL), Illinois Eye Bank (Chicago, IL) and National Disease Research Interchange (Philadelphia, PA). The project had the approval of the Institutional Review Board (IRB) Committee of University of California at San Francisco and Ethics Committee of Washington State University (Spokane, WA). All specimens were fixed in 10% buffered formalin, processed, and embedded in paraffin. Sections (5 μm thick) were prepared for hematoxylin and eosin (H&E) staining, immunohistochemistry, and TUNEL assay. The number of eyes examined at each stage is shown in Table 1.

**Table 1:** Number of eyes examined at each stage.

**Stage**	**Number of eyes**	**Stage**	**Number of eyes**
F7w	2	F21w	4
F8w	1	F22w	1
F9w	3	F23w	3
F10w	2	F27w	1
F11w	4	P5m	1
F13w	2	P11m	1
F15w	2	24y	1
F18w	1	48y	1
F19w	1	67y	1

F, fetal; w, week; P, postnatal; m, month; y, year.

Histological change during development of the anterior chamber angle was evaluated by light microscopy using hematoxylin and eosin stained sections. In addition, anti-CD68 antibody was used to label macrophages. Paraffin-embedded sections of the human liver were used as a positive control. Prior to primary antibody incubation, an antigen retrieval method was used on deparaffinized sections (boiled 10 mM sodium citrate buffer for 20 min, pH 6.0; Thermo Fisher Scientific, Fremont, CA). The sections were then incubated with monoclonal mouse anti-CD68 antibody (KP1, 1:50; Santa Cruz Biotechnologies, Santa Cruz, CA) overnight at 4 °C. Biotinylated donkey anti-mouse IgG (1:500; Jackson ImmunoResearch, West Grove, PA) was used as a secondary antibody at room temperature for 45 min followed by incubation with dichlorotriazinyl aminofluorescein (DTAF)-conjugated streptavidin (1:500; Jackson ImmunoResearch) for 30 min.

Detection of apoptotic cell death (TUNEL assay) was performed using DeadEnd^TM^ Fluorometric TUNEL System (Promega, Madison, WI) according to the manufacturer’s protocol. Fetal and adult sections treated with DNase I  (Sigma Aldrich, St. Louis, MO) before the assay were used as positive controls.

Sections stained with H&E were mounted in Permount^®^ (Thermo Fisher Scientific, Waltham, MA) and the others with aqueous mounting medium with 4',6-diamidino-2-phenylindole (DAPI; Vectashield; Vector, Burlingame, CA). Sections were observed under a fluorescence microscope (Axioskop; Carl Zeiss Meditec, Jena, Germany), and micrographs for each section were captured with an AxioCam HRC camera (Carl Zeiss Meditec) using Zeiss Axiovision software 4.2. (Carl Zeiss Meditec). Both immunohistochemistry and TUNEL assay experiments were repeated twice.

## Results

### Histology

**Figure 1:**
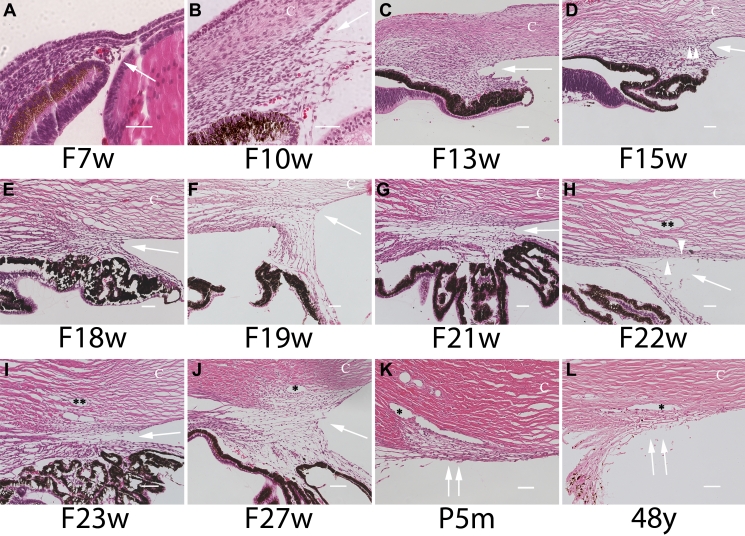
Photomicrograph pane showing histological development of the anterior chamber angle. At F7w (**A**), the angle structures are absent. At F10w and F15w (**B** and **D**), the primitive anterior chamber angle was formed by densely packed cells. At F18w and beyond (**E**), the cells at the iridocorneal angle decreased in number and density. Between F15w and F27w (**D**–**J**), the gradual appearance of immature trabecular beams with sparse intertrabecular spaces and lined by spindle shaped trabecular meshwork cells was noted. Note the anteriorly inserted ciliary body and incompletely cleaved angle from F7w to F27w. Schlemm’s canal appeared at F21w (**G**). A structurally mature Schlemm’s canal and a well developed trabecular meshwork was observed at F27w (**J**) and beyond, and a well developed trabecular meshwork was observed at P5m (**K**) and in the adult eye (**L**). F, fetus; w, week; m, month; y, year; C, Cornea; P, postnatal. The asterisk indicates the location of Schlemm’s canal. An arrow indicates an iridocorneal angle. Double arrows indicate the trabecular meshwork. Scale bar=50 μm.

At 7w of fetal life (F), the anterior chamber angle structures were absent in the fetal eyes examined. At F10w and F13w, a densely packed mass of mesenchymal/neural crest cells formed the primitive iridocorneal angle. Cellular attrition was noted in the fetal iridocorneal angle around F15w, and the angle structures became better delineated between F15w and F27w. The ciliary body was inserted anteriorly with mesenchymal/neural crest derived tissue in an incompletely cleaved angle. Immature trabecular beams with few intertrabecular spaces began to appear, and reduced cellularity was gradually observed. Precursors to Schlemm’s canal and spindle-shaped trabecular meshwork cells appeared at F22w. At F27w, a structurally mature Schlemm’s canal and a well developed trabecular meshwork was observed. The ciliary body was more posteriorly located with a well formed iris and partially cleaved angle (Figure 1). No morphological evidence of pyknotic nuclei, cellular debris, or inflammatory cells was seen in the areas of the developing angle in the sections examined.

### Immunohistochemistry

**Figure 2:**
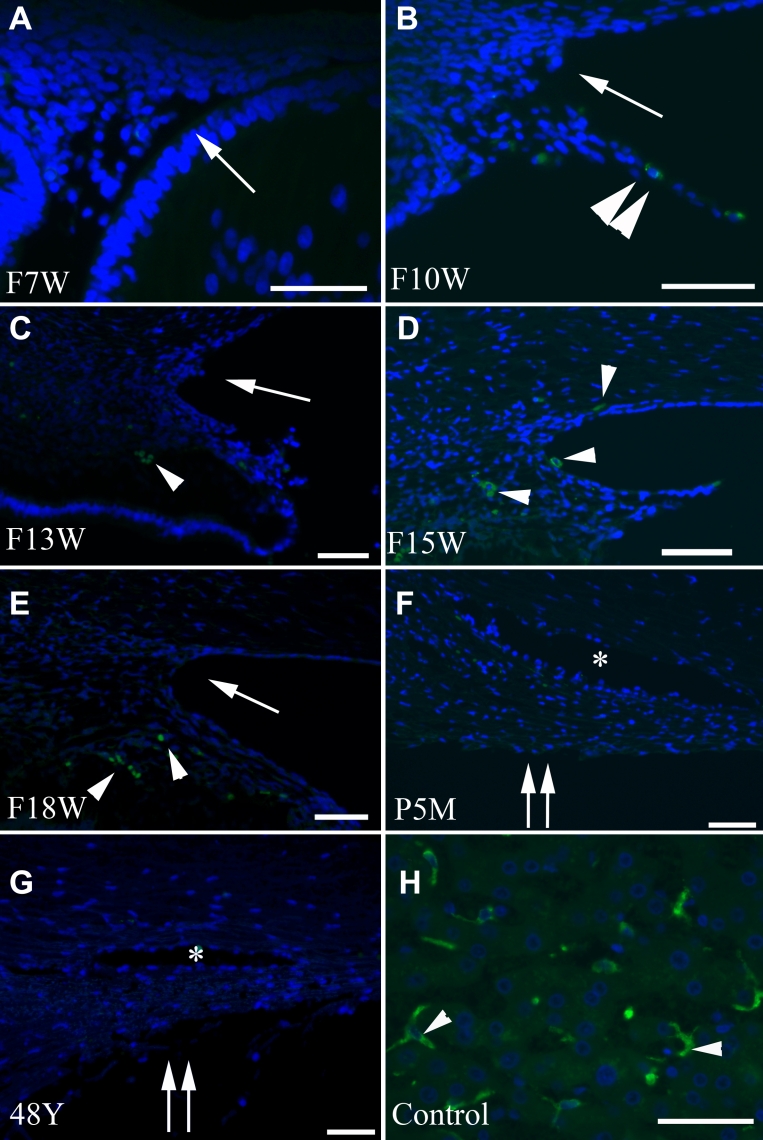
Photomicrograph panel showing CD68 immunostaining visualized with DTAF and nuclei counterstained with DAPI. The developing trabecular meshwork and surrounding areas do not show CD68 positive cells (**A**–**G**). Few CD68 positive cells, labeled in green, were observed in the iris stroma and anterior ciliary body from F10w through F18w (**B**–**E**; arrowhead) and absent at later stages (**F** and **G**). Note positive CD68 staining (**H**) in the human liver dendritic cells (arrowhead). F, fetus; w, week; m, month; y, year; P: postnatal. The asterisk indicates the location of Schlemm’s canal. An arrow indicates an iridocorneal angle. Double arrows indicate the trabecular meshwork. Scale bar=50 μm. Panel **D** is enlarged in Figure 3A to clearly show positively stained macrophages. Eyes from other fetal or adult tissues/stages that are not shown in the panel did not show CD68 positive macrophages.

**Figure 3:**
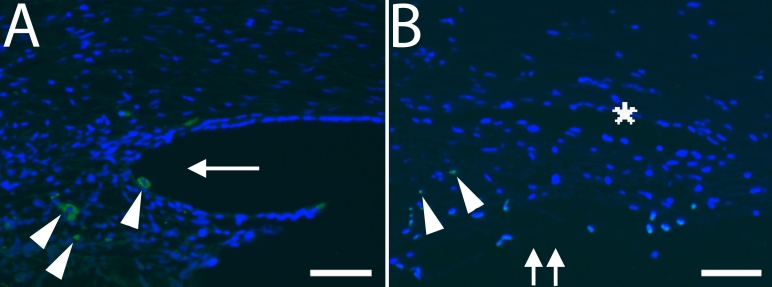
Immunohistochemistry and TUNEL assay. **A**: Magnified image of CD68 immunostaining at F15w from Figure 2. Note the cytoplasmic staining of cells in the developing ciliary body (arrowhead). The other green stain within the nucleus above is artifact. F, fetus; w, week. An arrow indicates an iridocorneal angle. Double arrows indicate the trabecular meshwork. Scale bar=50 μm. **B**: Magnified image of TUNEL staining in 48-year-old adult eye from Figure 4. Note the TUNEL positive nuclei that appear double stained with DAPI (arrowhead) in the trabecular meshwork, which is indicated by an asterisk. Double arrows indicate the trabecular meshwork. Scale bar=50 μm.

Positive CD68 immunofluorescence was not detected in cells that lined the iridocorneal angle and developing trabecular meshwork at any stage during development (Figure 2). Rare CD68-positive cells were transiently observed in the iris stroma and the developing ciliary body in fetal eyes that were 10 and 18 weeks old (Figure 2, Figure 3A). Positive immunolabeling was detected in dendritic Kupffer cells of the human liver, which was used as a positive control.

### TUNEL Assay

**Figure 4:**
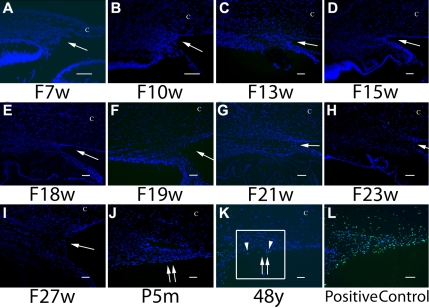
Photomicrograph showing TUNEL assay. TUNEL positive nuclei were visualized with fluorescein (green). Note the absence of TUNEL labeled nuclei in the anterior chamber angle in all fetal and infant eyes. Rare positively labeled cells were detected in the 48-year-old adult trabecular meshwork (**K**; arrowhead). Positive green nuclear staining was detected in the DNase I treated positive control (**L**). F, fetus; w, week; m, month; y, year; C, Cornea. An asterisk indicates Schlemm’s canal. An arrow indicates an iridocorneal angle. Double arrows indicate the trabecular meshwork. Scale bar=50 μm. The area enclosed in the box is enlarged in Figure 3B. Eyes from other fetal or adults tissues that are not shown in the panel did not show TUNEL labeling.

TUNEL labeled nuclei were not detected in the anterior chamber angle in any of the fetal or infant eyes examined (Figure 4). In an adult eye (48 years old), a few TUNEL positive cells were detected in the trabecular meshwork (Figure 3B). The size of the TUNEL positive cells appeared to be smaller than that of DAPI stained nuclei of surrounding cells, suggesting nuclear shrinkage of TUNEL positive cells. Positive TUNEL reaction was observed in the positive control treated with DNase I.

## Discussion

Abnormal development of the anterior chamber angle is frequently associated with elevated intraocular pressure, which can be a factor in the development of congenital glaucoma [2]. A study of the uncertain pathophysiological mechanisms underlying congenital glaucoma necessitates a strong conceptual understanding of the normal development of the anterior chamber angle, specifically the trabecular meshwork. Previously published theories of intertrabecular space formation generally fall into one of two different theories, either cell differentiation/reorganization or cell death/resorption.

In our study, we observed that the structural development of the human anterior chamber angle appeared consistent with previous reports [4-7]. There appeared to be a decline in cell numbers and density at the iridocorneal angle with maturation of the trabecular meshwork. Morphological evidence of cell death/resorption, inflammation, or tissue debris was not seen at any stage of angle development. We also investigated cell death/resorption in the developing human anterior chamber angle by immunohistochemistry for CD68 and TUNEL assay but could not demonstrate macrophage infiltration or cell death by these methods. Remé and coauthors [8] detected marked cellular necrosis and the presence of macrophages via light and electron microscopy in the developing rat anterior chamber angle from postnatal days 5 to 60. These results suggested that cell death may play a key role in opening the spaces of Fontana and the intertrabecular spaces in rats. A subsequent study by Richardson and coauthors [9] reported an increase in the number of macrophage-like cells detected by light and electron microscopy in the developing cat anterior chamber angle from postnatal days 3 to 9. It was speculated that localized degeneration might occur during anterior chamber angle development in cats, and the presence of macrophage-like cells in the trabecular meshwork might provide a mechanism for the removal of degenerating cells. In our study, differentiation of trabecular beams with intertrabecular spaces and reducing cellularity in human angle was observed between F15w and F27w. However, evidence of cell death and resorption were not seen during the period. Our findings in human angle development are in contrast to the reports in the rat and cat [8,9].

The presence of macrophage-like cells in the development of the human trabecular meshwork has also been demonstrated using light and electron microscopy at all stages of angle development, although there was no evidence of necrotic cellular debris [4,5,11]. However, there have been other reports that refute the cell death/resorption theory by showing the absence of histological evidence of cellular death or the presence of macrophages in dogs [12], monkeys [13], and humans [6]. In addition, a recent study conducted on mice reported that neither cell death nor macrophages were found when using light and electron microscopy and cell death assays during anterior chamber angle development [10]. Lack of evidence for cell death and resorption in our study advocates for the theory of cell differentiation/reorganization rather than that of cell death/resorption in human anterior chamber angle development, although the number of human eyes and developing stages examined was limited.

We did observe few TUNEL positive nuclei in the adult trabecular meshwork and CD68 positive macrophages in the developing ciliary body. Apoptotic trabecular endothelial cells have been noted in trabecular meshwork cells under various conditions in culture and in the glaucomatous trabecular meshwork [14,15]. The presence of TUNEL positive nuclei in the adult human trabecular meshwork endothelium has not been described. However, it is not surprising that TUNEL positive cells were seen since age related attrition of trabecular endothelial cells has been well described [16]. In addition, we also observed rare, transient, CD68 positive macrophages in the developing ciliary body and iris. However, no macrophages were noted in proximity to the developing trabecular meshwork. These findings are consistent with the description of Loffler et al. who described macrophages near the anterior ciliary body folds by electron miscopy in fetal eyes at 12–22 weeks in gestational age [17].

In summary, the current study examined anterior chamber angle development in human eyes between the age of F7w and F27w. The structural development of the human anterior chamber angle appeared consistent with previous reports. However, there was no evidence of cell death or resorption in the developing anterior chamber angle seen by light microscopy, immunohistochemistry, and TUNEL assay. Further studies may be needed to confirm these findings in a larger sample of human fetal eyes.
